# Association between Enzootic Pneumonia-Like Lung Lesions and Carcass Quality and Meat pH Value in Slaughter Pigs

**DOI:** 10.3390/ani13132210

**Published:** 2023-07-05

**Authors:** Paulina Przyborowska-Zhalniarovich, Dominiek Maes, Iwona Otrocka-Domagała, Katarzyna Paździor-Czapula, Agnieszka Wiszniewska-Łaszczych, Marta Sołtysiuk

**Affiliations:** 1 Department of Veterinary Public Health, Faculty of Veterinary Medicie, University of Warmia and Mazury in Olsztyn, 10-719 Olsztyn, Poland; marta.soltysiuk@uwm.edu.pl; 2 Unit of Porcine Health Management, Department of Reproduction, Faculty of Veterinary Medicine, Obstetrics and Herd Health, Ghent University, 9820 Merelbeke, Belgium; dominiek.maes@ughent.be; 3 Department of Pathological Anatomy, Faculty of Veterinary Medicine, University of Warmia and Mazury in Olsztyn, 10-719 Olsztyn, Poland

**Keywords:** PRDC, swine, bronchopneumoniae, pork quality

## Abstract

**Simple Summary:**

The prevalence of respiratory diseases in slaughter pigs ranges from 19% to 74%. Despite all efforts made to reduce economic losses caused by this condition, respiratory diseases continue to be an important concern for swine herds worldwide. However, few studies have investigated the relationship between respiratory disease and pork quality. The general aim of this study was to investigate the association between the respiratory health of slaughter pigs from different farms and carcass and meat quality. The main findings of this research confirm the negative influence of respiratory diseases on pork quality, resulting in a weight and meatiness reduction and deviation in pH value from the norm. This implies that lung lesions in slaughter pigs negatively influence not only animal health, welfare, and performance but also carcass quality.

**Abstract:**

Although the prevalence of respiratory diseases in slaughter pigs ranges from 19% to 74% and continues to be an important concern for swine herds worldwide, only a few studies have investigated the relationship between respiratory disease and pork quality. The general aim of this study was to investigate associations between the prevalence and severity of enzootic pneumonia-like lesions in Polish slaughter pigs on different carcass and meat-quality characteristics at the animal and herd levels. The average prevalence of bronchopneumonic lungs with different degrees of lesions was 94.57%. The majority of lesions indicated the acute stage of enzootic pneumonia. Our results indicate a statistically significant interaction between the mean weight of carcasses depending on the extent of the lesions (*p* = 0.04) at the animal level. The correlation between meatiness and severity of lung lesions was r = −0.25 (*p* = 0.00). The correlation between the extent of lung lesions and pH_45_ value was r = −0.17 (*p* = 0.005) on the animal level and r = −0.63 (*p* = 0.017) at the herd level. This implies that lung lesions in slaughter pigs negatively influence not only animal health and welfare, but also carcass quality.

## 1. Introduction

Porcine respiratory disease complex (PRDC) is a chronic respiratory disease mainly affecting growing and finishing pigs, causing major economic losses in the pig industry worldwide [1,2]. Generally, the prevalence of respiratory disease ranges from 19% to 74% in pigs. This large variation may depend on the study population (country and characteristics of selected farms) and the lung lesion scoring system used [3,4,5,6,7,8,9,10,11,12,13,14]. Mycoplasma hyopneumoniae (M. hyopneumoniae), Pasteurella multocida (P. multocida), Actinobacillus pleuropneumoniae (A. pleuropneumoniae), porcine circovirus (PCV), swine influenza viruses (SIV), and porcine reproductive and respiratory syndrome virus (PRRSV) are the most important microorganisms involved in PRDC. Cranioventral pulmonary consolidation (CVPC) is one of the main pulmonary lesions found in pigs during abattoir inspection. M. hyopneumoniae is the primary etiological agent of enzootic pneumonia (EP) and plays an important role in PRDC [8,9,10,15,16,17]. Despite all efforts made to reduce economic losses caused by this pathogen, M. hyopneumoniae continues to be an important concern for swine herds worldwide [1,2,8,9,10,15,16,17]. In addition to having a negative effect on pig health, animals with CVPC affecting more than 10% of the lung area have a lower average daily weight gain (ADWG) (6 to 9.3%) during the fattening period and a higher feed conversion ratio (0.16 units) [1,2,18,19,20,21]. A reduction in the ADWG by 41.1 g corresponds with a prolonged grower–finisher period of 16.7 days up to 104.5 kg slaughter weight and lower economic return [8,9,10,15,16,17]. The negative impact on performance is likely due to the negative impact of lung lesions on the metabolism of liver and muscle tissue. Strong evidence of an association was found between the presence of lung lesions and certain blood parameters. There was an increased level in certain biochemical indicators such as blood lactate and glucose and plasma sodium, chloride, and albumin, as well as enzymes found in the greatest amount in the skeletal muscle and liver that are responsible for maintaining energy homeostasis and are indicators of muscle and liver damage, such as CK (creatine kinase) and LDH (lactate dehydrogenase) [22].

The most important function of meat inspection in an abattoir is an evaluation of the carcasses for suitability for human consumption, consequently protecting public health [23,24]. Abattoir examination is a good opportunity for monitoring pig health and welfare through macroscopic identification of subclinical pathologies that are not possible to evaluate with observation at the farm of origin. Moreover, lung lesions may be scored to provide relevant information to the farmer and the herd veterinarian. Many scoring methods based on manual and visual evaluation are in place for assessing lung lesions in swine at the abattoir [25]. The abattoir evaluation of lung lesions is useful for estimating the prevalence and severity of respiratory diseases in swine herds, detecting subclinical states, providing information about on-going respiratory problems, and assessing risk factors and vaccine efficacy at the farm level. Finally, and also importantly for public health, assessing lung lesions may be used to evaluate the influence of respiratory diseases on carcass and meat quality [11,23,26,27,28]. Depending on the country, lung lesions are major causes for lung condemnation and lower speed on the slaughter line, as more carcasses need to be trimmed. It has been reported that CVPC is responsible for about 50.0% of lung rejections [2].

To date, few studies have investigated the relationship between respiratory disease and pork quality, although a few of these studies have shown that increased severity of pneumonia results in animals with lower live weight at slaughter time and may cause negative effects on pork quality by leading to changes in pH value, water holding capacity, color, flavor, and cooking quality [20,29,30,31,32,33,34]. The effect on acid–base balance, in combination with metabolic alteration in organs and tissues, might especially have a negative impact on carcass and meat quality.

These studies involved a small number of animals that were raised in a small number of herds, and results were only considered at the animal level.

The general aim of this study was to investigate the association between the respiratory health of slaughter pigs and carcass and meat quality. More specifically, associations were investigated between the severity of EP-like lesions in Polish slaughter pigs and different carcass and meat-quality characteristics.

## 2. Materials and Methods

### 2.1. Study Sample

A cross-sectional study was carried out in one abattoir located in northeastern Poland with a slaughtering capacity ranging from 300 to 400 animals per hour. The study took place from January to March 2021.

**Figure 1:**
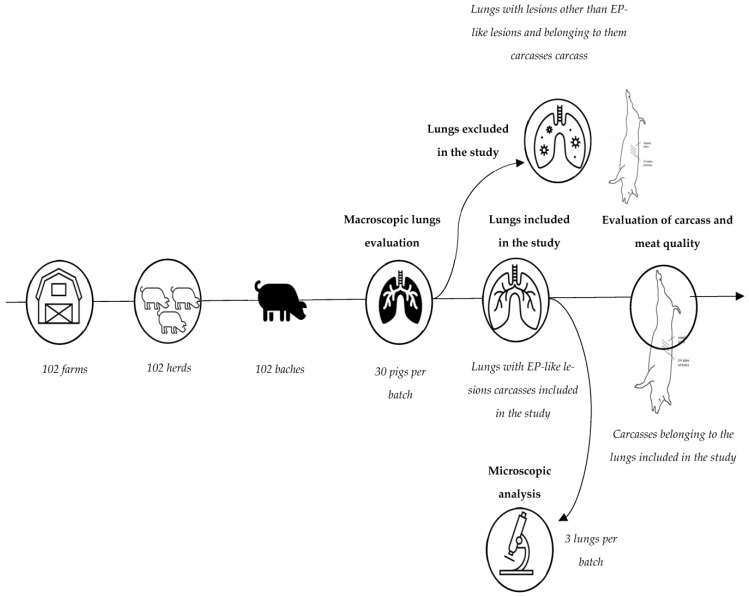
Description of the selection of the analyzed sample, depicting included and excluded subjects.

This study was conducted on pigs from 102 different, randomly selected herds from 102 different farms with similar housing conditions. All pigs included in this study were of the Polish Great White breed, weighing from 90 to 121 kg (105 kg on average), without sex distinction. From each herd, one batch of 120 pigs was selected. A batch was defined as a group of pigs belonging to the same farm that were killed on the same day at the abattoir. The total fasting period for all batches ranged from 12 to 15 h. For the examination of the lungs, carcass, and meat quality, as well as the analysis of histopathological lesions, 30 pigs from each batch were selected. Thirty pigs per herd were selected, as previous studies have shown that this number is recommended to obtain a reliable and accurate estimate of the severity of lung lesions at the herd level [10,35]. More specifically, the study sample consisted of a total number of 3060 lungs and carcasses from 102 batches from 102 herds. The selection of the study sample is presented in the flow diagram below (Figure 1).

### 2.2. Examination of Lung Lesions

#### 2.2.1. Macroscopic Lesions

The prevalence and severity of EP-like lung lesions were assessed by the first author and another trained veterinarian. The scoring results were obtained on two levels: animal and herd. The scoring method used to quantify EP-like lesions at the animal level was Madec and Kobisch (1982) and was related to individual pigs from each batch. The result for each animal is expressed as the sum of points for the assessed lung lobes according to Madec and Kobisch methodology (Table 1) [36].

**Figure 2:**
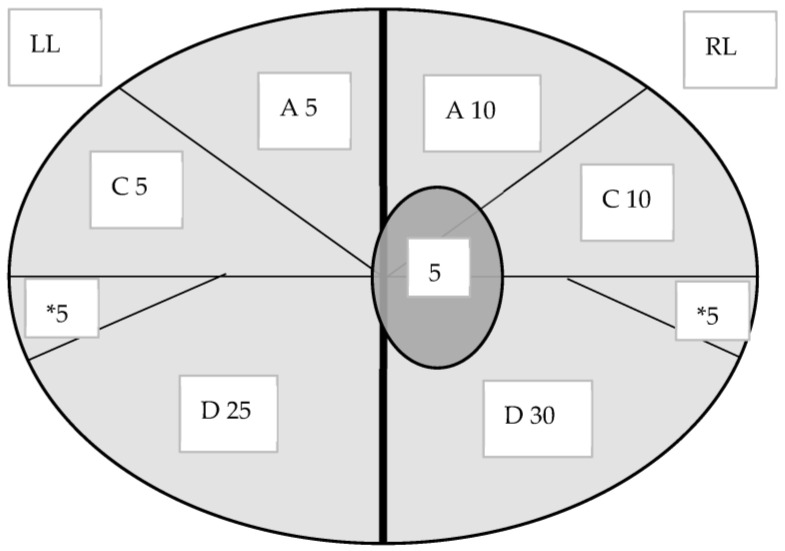
The enzootic pneumonia-like lesion scoring system according to Christensen methodology [37] used in the study. The percentage of each lobe considers—the volume it occupies in relation to the whole lungs. LL: left lung lobe, RL: right lung lobe, A: apical lobe, C: cardiac lobe, D: diaphragmatic lobe, *: cranial part of diaphragmatic lobe.

The scoring system used to quantify the lesions at the batch level was the software Ceva Lung Program (CLP), which is a digital calculating program dedicated to scoring lesions on lungs based on the combination of two scoring methods previously defined by Christensen et al. (1999) and Madec and Kobisch (1982) [36,37]. In the CLP system, each lobe is scored according to Madec’s scoring system, and the result of each lobe is then digitally normalized by its relative volume following the methodology reported by Christensen et al. (1999). The Christensen approach consists of estimating the percentage of each lung lobe affected area multiplied by the lobe relative weight and summed to provide the total weight percentage of affected lung. An extra score was given to the cranial area of the diaphragmatic lobes (Figure 2) [37]. We used special calculation software to express the lung score for the analyzed group.

The results at the batch level are expressed using CLP software as four parameters: percentage of bronchopneumonic lungs (%) (sum of lungs with lesions/sum of total examined lungs × 100%), a parameter expressing the prevalence of lesions in the examined population; the percentage of the affected surface in bronchopneumonic lungs (%), a parameter expressing the severity of lesions; the percentage of lungs in the chronic stage (%); and the enzootic pneumonia index (EP-Index). The EP-Index was calculated using software as the mean result of all scored lungs from each batch.

**Figure 3:**
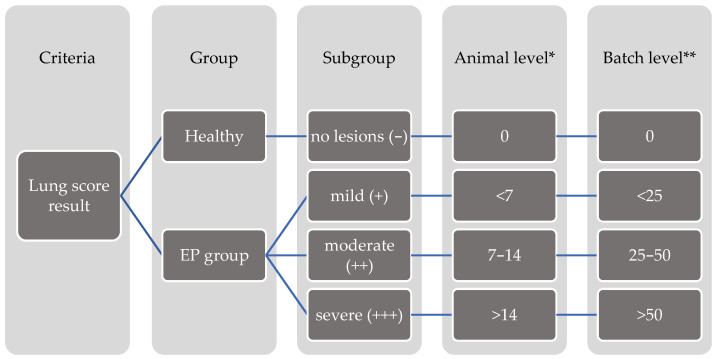
The schema of grouping on animal and batch level based on prevalence and severity of EP-like lung lesions. The grouping criterion was absence/presence of lesions (expressing prevalence of lesions at two levels). Subgrouping criterion was severity of lesions, expressed by lung scoring result at animal level and expressed by percentage of affected surface in bronchopneumonic lungs at batch level. – group of animals without EP-like lesions; + group of animals with mild EP-like lesions; ++ group of animals with moderate EP-like lesions; +++ group of animals with severe EP-like lesions. * Scoring units: points according to Madeck and Kobish grid, maximum total score, 28 points. ** Scoring units: percentage, according to CLP software, maximum total score, 100%.

Grouping was provided depending on the absence/presence of lesions and their severity for both animal and batch level (Figure 3). The absence of visible lung tissue disorders (including EP-like lung lesions, pleurisy, and other lesions) was classified as healthy (−), whereas the presence of EP-like lesions was described as the EP group.

Depending on the severity of EP-like lung lesions and level, the EP group was divided and classified into three subgroups and described as mild (+), moderate (++), and severe (+++) when lesions occupied <7, 7–14, and >14 of the lung surface at the animal level or <25%, 25–50%, and 51–75% of the lung surface at the batch level, respectively.

#### 2.2.2. Histopathological Lesions

To confirm that the lesions were indicative of EP, three lungs showing the most extensive EP-like lesions within each batch were selected for histopathological examination. Firstly, a lung section of approximately 5 g was collected at the margin of the lesion with the surrounding normal parenchyma from the apical and cardiac right lobe. As a control, analogous samples of lungs with no visible lesions were taken for further histopathology as well. The samples were fixed in 10% neutral buffered formalin for routine histopathological evaluation. The tissues were processed routinely in a tissue processor (Leica TP1020, Leica Biosystems, Deer Park, USA), embedded in paraffin, and cut at 5 μm. The initial sections were stained with hematoxylin and eosin (HE) and analyzed using a light microscope (Olympus BX43, Olympus, Tokyo, Japan). 

Additionally, the lesions were scored with microscopic grading criteria to investigate if there were any correlations between the results and macroscopic lesions. The scores could range from 0 to 5 according to the degree of peribronchiolar and perivascular lymphohistiocytic infiltration as well as cuffing formation (Villareal 2009, Morris 1995). Scores 1 (limited cellular infiltrates, macrophages, and lymphocyte, around bronchioles, with airways and alveolar spaces free of cellular exudates) and 2 (moderate infiltrates with mild diffuse cellular exudates into airways) were classified as lesions not related to mycoplasma infections. Scores 3, 4, and 5 were considered to be associated with mycoplasma infection (mild, moderate, or severe bronchointerstitial (cuffing) pneumonia, surrounding bronchioles but extending to the interstitium, with lymphofollicular infiltration and mixed inflammatory cell exudates). The average histopathological lung score per batch was calculated.

### 2.3. Carcass and Meat Quality Analyses

The carcass and meat quality of the 30 pigs that were selected for lung lesion scoring were evaluated. To this end, the carcass weight was assessed as well as the meatiness.

The carcass was weighed immediately after splitting and final washing to obtain the carcass weight (m). Meatiness was measured with a portable Sydel CGM Fat/Lean Sensor device (Syleps, Lorient, France) on the musculus longissimus dorsi, pars lumbalis, on the left half of the carcass, at the level of the 10th and 11th ribs in accordance with the applicable legislation (Regulation (EU) 2017/1182) [38]. Based on the meatiness result, the classification of carcass quality was scored following the EUROP scale (Table 2).

The pH_45_ value and temperature measurements were related to meat quality. A Hanna HI 99163 portable pH meter (Hanna Instruments, Smithfield, Rhode Island, USA) was used to measure initial meat temperature (T_I_) and pH (pH_45_) 45 min postmortem on the musculus longissimus dorsi, pars lumbalis, on the left half of the carcass, at the level of the 10th and 11th ribs. Following Cobanovic et al. (2017) [39], the carcasses showing pH_45_ values lower than 6.0 were classified as PSE (pale, soft, exudative) meat, whereas those with pH45 values higher than 6.4 were classified as DFD (dark, firm, dry) meat. Carcasses with a pH_45_ between 6.0 and 6.4 were classified as pork of normal quality [39].

In three pigs from every batch, a Testo 175T2 temperature data logger (Testo, Titisee-Neustadt, Germany) was inserted into the carcasses. The loin temperature data logger was placed in an incision made between the tenth and eleventh ribs. The dataloggers recorded the time and temperature at 1 min intervals. The data loggers were removed from the carcasses as the carcasses entered the cutting floor (approximately 24 h postmortem). The initial (T_I_) and ultimate (T_U_) temperature recordings were used for analyses.

### 2.4. Statistical Analysis

Statistical analysis was performed using Statistica 13 PL software. The Shapiro–Wilk test was used to examine the distribution of variables, and Levene’s test was used to determine the homogeneity in their variance. An analysis of variance (ANOVA) followed by multiple comparison tests was used to compare the differences between the mean values of carcass weight and lung scoring results at the animal and batch levels. The hypotheses about the correlations between variables such as lung scoring results, meatiness, and pH_45_ were verified using Pearson’s r correlation test. The level of statistical significance was assumed to be α = 0.05. The results were considered statistically significant if α < 0.05.

## 3. Results

### 3.1. Proportion and Severity of EP-Like Lesions

A summary of the results is presented in Table 3. Only 12 (11.76%) of the examined batches had no visible macroscopic lung lesions, whereas 46 (45.10%) batches displayed EP-like lesions. A total of 44 batches (43.14%) were excluded from further investigation due to the presence of pathological lung changes other than cranio-ventral pulmonary consolidation. Thus, 58 examined batches were taken for further investigation: 46 with different severities of EP-like lesions and 12 with no evidence of pulmonary lesions.

At the batch level, the average proportion of bronchopneumonic lungs with different degrees of lesions ranged from 90.93% to 95.48% (mean value = 94.57%). In the examined sample, the majority of lesions indicated the acute stage of enzootic pneumonia, ranging from 73.90% to 90.97%, whereas the percentage of lesions indicating the chronic stage ranged from 9.03% to 26.10%. Table 4 contains detailed data on lung scoring. The mean EP-Index for the EP group was 16.12, whereas the mean EP-Index for the healthy group was 0.98.

### 3.2. Histopathological Lesions

**Figure 4:**
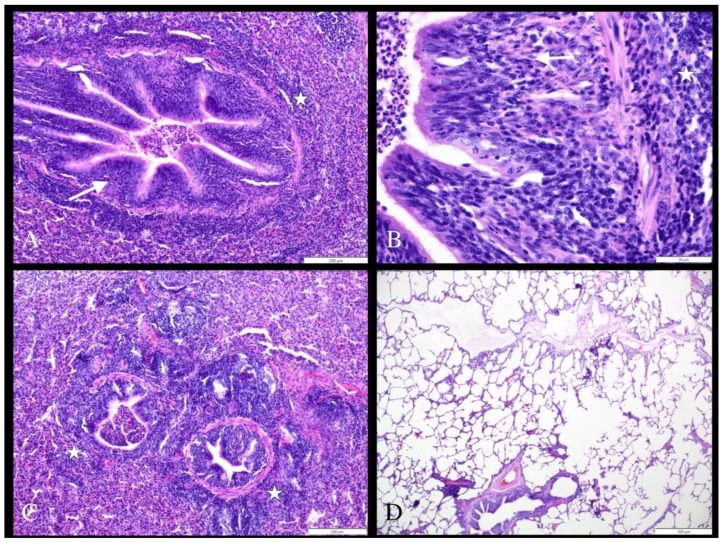
Histopathological findings in lung tissue samples. (**A**–**C**) Gross cranioventral pulmonary consolidation associated with enzootic pneumonia. There are distinct peribronchiolar inflammatory cell cuffs composed of macrophages, lymphocytes, plasma cells, and eosinophils (asterisks). The bronchial mucosa is thickened and infiltrated with variable numbers of inflammatory cells (arrows) (**A**,**B**). (**D**) Healthy lung tissue. No microscopic changes are observed. HE. Scale: 20 μm (**A**); 50 μm (**B**); 200 μm (**C**); 500 μm (**D**).

The histological findings show pathological changes typical for bronchopneumonia. In all samples with macroscopic EP-like lesions, i.e., the accumulation of exudate composed of eosinophils, less numerous macrophages, and neutrophils within the lumen of bronchi and bronchioles, the bronchial mucosa was variably infiltrated with lymphocytes, plasma cells, eosinophils, and macrophages. Distinct peribronchial and peribronchiolar inflammatory cell cuffs were also observed. The bronchus-associated lymphoid tissue (BALT) was hyperplastic. The histological findings indicate enzootic pneumonia (Figure 4A–C).

In all control samples, only healthy lung tissue (no histopathological lesions) was found. The average score of the microscopic lesions was 4.3 (SD = 0.4), but there was no significance or difference between results in subgroups with lesions (*p* = 0.13) and no significant correlation between macro and microscopic scoring results, r = 0.21 (*p* = 0.27).

### 3.3. Carcass and Meat Quality

Table 5 gives an overview of the mean values of carcass and meat quality traits according to lung lesion severity.

The parameters of T_I_ and meat pH show a low range of variation (maximum–minimum) in all groups. For data analyzed at a batch level, there was a significant negative effect of the severity of lung lesions on the weight and pH_45_. Batches with a moderate-to-severe EP-Index had a significantly higher differentiation in carcass weight compared to those with lower EP-Index scores. The development of meat quality abnormalities in the evaluated sample with EP-like lung lesions was high (68.3%, on average). In batches with mild EP-like lesions, 80% of cases had pH_45_ > 6.4, which increased the tendency towards DFD meat. In the examined population, among carcasses with a mild EP-Index, there were no cases with pH_45_ < 6.0. In batches with a higher EP-Index, the tendency towards DFD meat was lower (in 42.1% and 36.0% for batches with moderate and severe lesions, respectively). Carcasses with moderate and severe lesions had a greater tendency towards PSE meat (26.3%, and 20.0%, respectively) than those with mild lesions.

The mean initial temperature in all groups was 40.6 °C (40.5–40.6 °C). Neither the initial carcass temperature nor the temperature decline was influenced by the proportion or severity of EP-like lung lesions. In all cases, carcasses reached their ultimate temperature after 24 h of chilling without any deviation in temperature decline.

**Figure 5:**
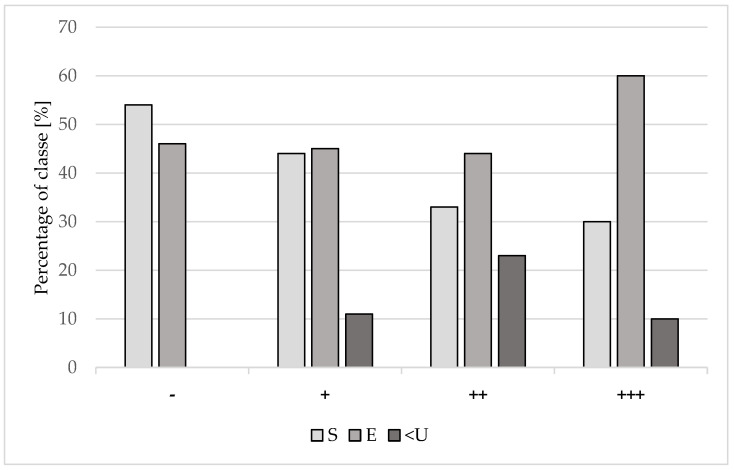
Percentage of carcass classes in 1.740 carcasses. The bar chart shows the percentage of the individual carcass classes on the EUROP scale in batches with different extensions of lung lesions. −: batches with no evidence of EP-like lung lesions; +: batches with mild EP-like lesions; ++: moderate EP-like lesions; +++: severe EP-like lesions. S, E, U: classification notes in EUROP scale.

The classification of carcasses based on meatiness according to the EUROP scale for all investigated groups is presented in Figure 5. According to the results, in subgroups with EP-like lung lesions, the class of meat was downgraded to under U-class in only 10–23% of carcasses. A detailed description of pH_45_ changes in individual groups involving carcass classification is shown in Table 6.

### 3.4. Associations between Lung Lesions and Carcass/Meat Quality

A statistical analysis showed that there are significant differences in the mean weight of carcasses among the groups of lung lesions at the animal level (*p* = 0.04). Significant differences in average carcass weight were found between the healthy and EP-group, whereas at the batch level, significant differences between these groups were lower (*p* = 0.41). The post hoc test showed only a statistical difference between the average carcass weight and the extent of the moderate and mild changes at the animal level (*p* = 0.02).

The correlation between meatiness and severity of lung lesions was r = −0.25 (*p* = 0.00). The correlation between the extent of lung lesions and pH_45_ value was r = −0.17 (*p* = 0.005) at the animal level and r = −0.63 (*p* = 0.017) at the herd level.

## 4. Discussion

The main findings of this research confirm the negative influence of respiratory disease on pork quality, resulting in a weight and meatiness reduction and deviation in the pH value from the norm.

In the present study, the assessment of lung lesions revealed a high proportion of EP-like lung lesions in the examined pigs (45.10%). This study was conducted on a group of 3060 pigs from 102 different farms with similar housing conditions to obtain a reliable assessment of the proportion and severity of respiratory diseases in the sample population. This proportion is likely higher than in the average population, as the selected farms had clinical signs of respiratory disease. The prevalence of pigs with different degrees of lung lesions was comparable to results reported by other authors in Europe [3,4,5,6,7,8,9,10,11,12,13,14,40]. However, it is challenging to relate such data between reports because of the several sources of variation that exist [41], and the high prevalence of documented conditions may indicate a need for improvements in the control and further monitoring of the investigated diseases in Polish pig herds.

Compared with other entries in the literature, a histopathological examination revealed similar microscopic changes in all samples such as epithelium hyperplasia, cilia density reduction, peribronchiolar and perivascular lymphohistiocytic infiltration, and cuffing formation [42]. In some papers, the microscopic scoring was provided together with macroscopic lung scoring to quantitatively assess the effects of changes in experimentally inoculated swine [43,44,45,46] but in field samples, as long as changes are observed, this approach may be less applicable.

This study reports that the mean weight of carcasses depended on the extent of the lesions. The results vary between levels. At the batch level, the influence on weight was not significant, whereas there was a noticeable difference in carcass weight at the animal level. Several studies have reported that the presence of severe lung lesions in slaughter pigs resulted in reduced live weight and carcass weight [30,47]. This could be attributed to the fact that growth rate, feed conversion, and daily weight gain during the fattening period are lower in pigs with respiratory lesions [19]. Consequently, these metabolic alterations induce a reduction in body weight as well as significantly downgrade carcass quality [34,48]. Contrary to previous research, the current study indicates that the difference in weight in carcasses affected with pulmonary lesions was statistically significant only in pigs with mild and moderate lung lesions. On the other hand, in batches affected with severe EP-like lung lesions, the difference between carcasses was over 1/3 of the carcass weight, and the variation in the population was 20.1%. This could suggest that for those batches, the fattening period may have been extended. Unfortunately, the authors did not have detailed information about the age of the slaughtered animals. Otherwise, the analyzed relationship between the extent of changes in the lungs and the weight of the carcass would be more accurate.

From the association found between meatiness and lung lesion score, it was possible to calculate the meatiness losses that these animals had at slaughter due to respiratory disease. Interestingly, this study reported that for each increase of one percent in the lesion area, there was a decrease of 0.24% in meatiness. Consequently, in pigs with lung lesions, a reduction in the carcass quality class was observed more frequently. In this study, pigs show a depressed meatiness and a reduced meat class below U (on the EUROP scale) in 10–23% of cases. Comparable results were reported by Cobanovic et al. (2021), who found that pigs with severe lung lesions produced the lowest percentage of E class carcasses and the highest percentage of P class carcasses.

In the current study, neither the appearance nor the severity of EP-like lung lesions influenced the initial carcass temperature or temperature decline. The data show that there is evidence of an association between the presence of lung lesions in slaughtered pigs and changed meat pH, which led to the highest occurrence of DFD and PSE pork. A statistical analysis revealed that for each increase of one percent in the lesion area, there was a decrease of 0.0021 in pH_45_. The meat quality results obtained in this study are in agreement with Karabasil et al. (2017) and Cobanovic (2021), who reported that the risk of obtaining pork abnormalities (PSE and DFD) was higher in pigs showing severe lung lesions. The change in initial temperature postmortem is an important factor in the meat industry. Adequate carcass chilling is required to optimize pork quality and food safety. Moreover, the initial and ultimate pH values, together with the temperature, have a critical role in defining and maintaining pork quality. It is known that there is a high possibility for initial low pH and a high-temperature postmortem to induce heat shortening and PSE meat. However, in the case of the loss of glycogen due to long-term stress, the pork is unable to produce lactic acid and instead demonstrates characteristics, termed DFD, that maintain a high ultimate pH. Meat quality is described by the sum of all meat-quality characteristics, which are typically adjusted by the effects of muscle pH [49,50,51,52,53,54,55].

Although the study population in the current research involved 3066 pigs from 102 different batches representative for the same number of herds and farms, the authors are aware of two major limitations of this study. The first limitation is a lack of knowledge about herd health and management, since various factors have an influence on pork quality, such as genetic variables, feeding, slaughter weight, and sex, as well as preslaughter and slaughter conditions [23,32,34,39,52,53,56,57,58]. The second limitation is a restricted analysis of meat characteristics focusing only on basic quality parameters. Therefore, slaughterhouse inspection alone is insufficient. For a more accurate assessment of the impact and significance of respiratory diseases on meat quality, a further examination is needed. Detailed analyses related to the biochemistry of meat could lead to a clearer link between the impact of respiratory diseases and meat quality.

## 5. Conclusions

The present study found that the presence and extent of lung lesions were associated with carcass quality, expressed as meatiness, but were not strongly associated with carcass quality, expressed as carcass weight. However, the extent of lung lesions was negatively associated with meat quality, as it increased the risk for PSE and DFD meat. This implies that lung lesions in slaughter pigs not only negatively influence animal health, welfare, and performance, but also meat quality. Further research on the possible influence of farm characteristics and genetics of the animals on this association is warranted.

## Figures and Tables

**Table 1:** The enzootic pneumonia-like lesion scoring system according to Madec and Kobisch methodology [36] used in the study.

Enzootic Pneumonia-Like Lesions (per Lobe)	Score *
No lesions	0
Lesion affecting <25% of the lobe surface	1
Lesion affecting 25–49% of the surface	2
Lesion affecting 50–74% of the surface	3
Lesion affecting >75% of the surface	4

* Scoring units: points, maximum total score of 28 points.

**Table 2:** The pig carcass classification scale.

Weight Seurop Code	Description (% of Lean Meat)
S	60 meat or more
E	55 to less than 60
U	50 to less than 55
R	45 to less than 50
O	40 to less than 45
P	less than 40

**Table 3:** Summary of the study sample results.

Nr of Farms/Herds/Batches	Nr of Pigs	Nr of Examined Lungs	Nr of Lungs Affected by EP-Like Lesions	Nr of Lungs Excluded from the Investigation	Average EP- like Lesion Score *	Nr of Lungs in Groups				Nr of Lungs with Histopathological Analysis	Nr of Examined Carcasses
						Healthy	EP-Group				
							+	++	+++		
102/102/102	3060	3060	1364	1320	12.6	376	298	707	359	306 *	1740 **

* The samples for histopathological analysis were collected from 3 lungs of each set of 30 lungs selected per batch for macroscopic evaluation. Samples were not collected from lungs excluded from the investigation. ** When lungs were excluded from investigation, carcasses belonging to them were excluded as well. +: batches with mild EP-like lesions; ++: moderate EP-like lesions; +++: severe EP-like lesions.

**Table 4:** Average results of lung scoring for the examined sample.

Lung Lesion Severity	Number of Herds	Bronchopneumonic Lungs/Total Lungs (%)	Affected Surface/Bronchopneumonic Lung (%)	Lungs with Scars/Total Lungs (%)	EP-Index
−	12	16.67	2.79	3.1	0.98
+	10	90.93	22.19	26.10	10.81
++	24	95.48	33.36	22.94	15.88
+++	12	95.48	51.83	9.03	21.53

Data defined for the batch can be interpreted at both animal and batch levels. The percentage of bronchopneumonic lung indicates the proportion of EP-like lung changes in batches related to the appearance of lesions in an animal. A high value in a subgroup indicates that the population in each is homogenous for lesions. The percentage of the affected surface represents the extension of changes in a batch and can be considered at the animal level as well as at the batch level. – group of animals without EP-like lesions; + group of animals with mild EP-like lesions; ++ group of animals with moderate EP-like lesions; +++ group of animals with severe EP-like lesions.

**Table 5:** The descriptive data of investigated mean values for carcass and meat.

		Lung Lesion Severity			
		−	+	++	+++
	Average EP-Index	0.98	10.81	15.88	21.53
	Number of herds	12	10	24	12
	Percentage of herds (%)	11.8	9.8	23.5	11.8
Carcass weight	m (kg)	95.3	98.2	95.9	98.9
	SD (kg)	5.0	7.1	7.7	10.5
	V_m_ (%)	5.2	17.4	18.5	20.1
	Min.–max. value (kg)	90.1–103.7	86.3–112.3	81.7–114.3	82.5–119.2
Meat pH	pH_45_	6.2	6.5	6.2	6.3
	SD	0.2	0.3	0.3	0.3
	V_pH_ (%)	3.2	4.6	4.8	4.8
	Min.–max. value (%)	6.0–6.4	6.1–6.9	5.6–6.6	5.8–6.9
Meat temperature	T_I_ (℃)	40.6	40.5	40.5	40.6
	SD (℃)	0.6	0.9	0.6	0.5
	V_T1_ (%)	0.2	0.5	0.3	0.5
	Min.–max. value (%)	39.5–41.9	39.6–41.7	39.1–41.4	39.9–41.4
Carcass meatiness	Meatiness (%)	60.0	58.7	57.9	58.8
	SD (%)	1.6	3.8	2.8	2.2
	V_Meatiness_ (%)	6.3	18.9	18.9	21.1
	Min.–max. value (%)	57.6–63.2	49.9–62.5	53.3–61.1	54.8–61.7

Batches with no (−), mild (+), moderate (++), and severe (+++) EP-like lung lesions; m: mean carcass weight; SD: standard deviation; V: variation; min.- max.: minimum to maximum value; TI: mean initial temperature value, measured 45 min postmortem; pH45: mean pH value measured 45 min postmortem.

**Table 6:** pH_45_ changes involving carcass classification.

EP Group	EP-Index	pH_45_ < 6.0		Meat Classification (Europ)		
		% of Carasses	Mean pH_45_	S	E	≤U
+	10.81	0%	0	0%	0%	0%
++	15.88	26.3%	5.7	0%	60.0%	40.0%
+++	21.53	20.0%	5.8	40.0%	40.0%	20.0%
		pH_45_ > 6.4				
+	10.81	80.0%	6.7	50.0%	37.5%	12.5%
++	15.88	42.1%	6.6	12.5%	87.5%	0%
+++	21.53	36.0%	6.8	44.0%	56.0%	0%

The table contains information on the percentage of carcasses with deviations in pH_45_ value from the norm (6.0–6.4), taking into account the EUROP-scale carcass classification (included S, E, and U note) and the percentage of carcasses categorized into a given class for groups with pH_45_ deviations. +: batches with mild EP-like lesions; ++: moderate EP-like lesions; +++: severe EP-like lesions.

## Data Availability

The data presented in this study are available on request from the corresponding author.
